# Impact of two brief behavioral theory–driven professional training programs on fitness center attendance: protocol for pragmatic controlled intervention with random allocation

**DOI:** 10.3389/fpsyg.2026.1856891

**Published:** 2026-07-02

**Authors:** Diogo S. Teixeira, Panteleimon Ekkekakis, Hassan Mohamed Elsangedy, Vasco Bastos

**Affiliations:** 1 Faculty of Physical Education and Sport, Lusófona University, Lisbon, Portugal; 2 Research Center in Sport, Physical Education, Exercise and Health (CIDEFES), Lisbon, Portugal; 3 Centre for Research, Training, Innovation and Intervention in Sport (CIFI2D), Porto, Portugal; 4 Michigan State University, Michigan, MI, United States; 5 Department of Physical Education, Federal University of Rio Grande do Norte, (UFRN), RN, Brazil

**Keywords:** adherence, exercise, fitness, hedonism, motivation, self-determination

## Abstract

Fitness centers are privileged settings for promoting health; however, they are affected by high attrition rates and low attendance. At the same time, several motivational theories are considered relevant to improving behavior in exercise contexts, although they largely lack experimental evidence. Accordingly, the main objective of this study is to examine attendance trajectories following the implementation of two short training programs for exercise professionals, based on two theories of human behavior (self-determination theory and hedonic theory), with fitness center attendance patterns over time. As a secondary objective, the study will examine associations with attrition rates, motivational variables, and other behavioral outcomes. It is hypothesized that the theory-driven interventions will be associated with more favorable attendance trajectories and lower attrition than the control condition. This is a pre-registered, pragmatic, single-blind, controlled intervention study with three parallel arms, in which fitness clubs were randomly allocated across conditions. Professionals will receive brief training sessions according to their assigned condition throughout the intervention period. From September 2025 to August 2026, data on dropouts and training attendance among new clients will be collected. A subsample of exercisers will also be followed longitudinally to assess motivational outcomes. Primary outcomes will be attendance over one year; secondary outcomes will include attrition rate, motivation, intention to continue exercising, behavioral automaticity, subjective affective experience, and enjoyment. Improvements in attendance would reflect individual health gains, while reductions in attrition rates would enhance the financial sustainability of clubs. Moreover, the findings may provide preliminary evidence regarding the practical utility of translating these theoretical approaches into routine professional practice.

**Clinical trial registration:**

ClinicalTrials.gov, identifier NCT07156240.

## Background and rationale

Globally, the estimated number of individuals participating in fitness center activities exceeds 200 million (International Health, Racquet, and Sports Club Association, 2020), making the development of methods to support exercise adherence in the context of fitness centers important from a public-health standpoint. The presence of qualified exercise professionals not only has the potential to enhance the fitness and health benefits of exercise but may also play a crucial role in promoting physical activity as a sustainable lifestyle choice. Given this, it is concerning that fitness centers have often been overlooked by researchers and policymakers, highlighting a lack of attention and investment in studying and promoting regular exercise in these settings (Sperandei et al., 2016). Nonetheless, some scholars in the fields of behavioral science and motivation have been exploring the complex array of factors that influence motivation to initiate and maintain fitness routines.

Leading theories of human behavior, including the Theory of Planned Behavior (Ajzen, 1991), the Self-Determination Theory (Deci and Ryan, 1985), and the Transtheoretical Model (Prochaska and DiClemente, 1982), have been applied across various physical activity (PA) settings, yielding mixed results. While they have provided some valuable insights, theory-based interventions have not yet consistently produced evidence that they could be scaled to foster sustained, population-wide increases in PA (Ekkekakis and Zenko, 2016; McEwan et al., 2019; Pratt et al., 2020; Rhodes et al., 2021). This lack of significant progress has prompted frustration and a search for new perspectives, as these established theories, despite some support for their postulates, have not translated into meaningful and lasting behavior change. Some authors have criticized the theories for their reliance on a narrow set of cognitive appraisals (e.g., supportive social environments; benefits vs. barriers; self-efficacy) (Conn et al., 2011; Ekkekakis, 2017; Rhodes et al., 2019). Other critics have pointed out an overwhelming preponderance of correlational studies and a relative lack of experimental tests of theoretical postulates (Ekkekakis et al., 2019; Manninen et al., 2022; Ntoumanis and Moller, 2025). To overcome these limitations, new theoretical approaches and experimental studies are urgently needed (Sheeran et al., 2025; Simpson et al., 2025).

To this end, the present study will address the issue of maintaining PA behavior through an experimental approach using two theoretical perspectives. The first perspective is grounded in a well-established theory of human motivation, the Self-Determination Theory (SDT), which offers several specific predictions regarding variables that can be manipulated to enhance exercise adherence. The second approach is structured in alignment with hedonic theory, a recently revitalized approach to understanding human behavior, focusing on the promotion of pleasurable responses during exercise.

### Self-determination theory as a potential framework to support exercise attendance

Of the several motivational theories and models developed over the years, SDT presents a very appealing framework that could be operationally applied in various PA contexts, such as fitness centers. SDT is a macro-theory of human behavior that encompasses several subtheories. Of particular relevance to the aims of this study are the basic psychological needs (BPN) and the organismic integration subtheories. The former posits that the needs for autonomy (the sense of being in control of one’s actions), competence (the perceived ability to succeed at desired levels of challenge), and relatedness (the establishment of deep, meaningful interpersonal relationships with others) are essential psychological nutrients for personal growth and development. These needs are presented as essential precursors to the quality of motivation and, subsequently, several positive outcomes, such as continuous engagement in various activities (Ryan and Deci, 2017; Ntoumanis et al., 2021). The organismic integration subtheory relates to the concept of motivational quality, which emerges within the individual over time as a reflection of the organismic assumptions. The transition from extrinsic to intrinsic forms of motivation is theorized to be facilitated by the satisfaction of BPN, leading, if performed successfully, to more autonomous expressions of the self, which is believed to be key for the promotion of well-being and, of interest to this work, for supporting sustained positive behavior change (Ryan and Deci, 2017; Ryan et al., 2022).

To date, relatively few experimental tests of this theory focusing on behavioral outcomes have been conducted in PA settings, and even fewer in fitness contexts. Of several recent meta-analyses that included studies using the theory in PA [e.g. (Manninen et al., 2022; Ryan et al., 2022; Gillison et al., 2019; Ng et al., 2012)], surprisingly few focused on adherence or exercise session attendance, an unexpected finding given the 40-year history of the theory. Moreover, the few available studies have demonstrated heterogeneous small-to-medium effects of theory-driven interventions (Manninen et al., 2022; Ntoumanis and Moller, 2025), highlighting the need for more experimental research (Manninen et al., 2022; Moustaka et al., 2012; Ntoumanis et al., 2017), longer interventions (Moustaka et al., 2012; Edmunds et al., 2008; Fenton et al., 2021), co-acting strategies (Gillison et al., 2019), and the establishment of synergies across different theoretical frameworks (Arey et al., 2022; Ntoumanis et al., 2018).

Overall, as evidenced by its popularity, SDT is seen as highly relevant to the context of PA and holding great promise. At the same time, however, the promise has yet to fully materialize by establishing robust evidence of improved behavioral adherence. The present experimental study is aimed at helping strengthen this evidence base.

### The hedonic theory and its role in (automatic) motivation

Psychological or motivational hedonism refers to the doctrine that behavior is driven by the desire to attain pleasure and avoid displeasure. Although the pursuit of hedonic pleasure is clearly not the sole determinant of human motivation, it is regarded as an underrecognized element in the study of PA motivation (Ekkekakis and Dafermos, 2012). Despite a shortage of high-quality experimental research, the growing dissatisfaction with cognitive theories and advocacy for alternative or multi-theoretical frameworks have renewed interest in hedonic-based motivation in the context of PA (Ekkekakis, 2017; Ntoumanis et al., 2018; Dukes et al., 2021).

Embedded within hedonic assumptions is the concept of the affect heuristic. This concept reflects a basic hedonic premise: affect tends to guide behavior by facilitating decisions that are likely to increase pleasure and decrease displeasure (Ekkekakis and Zenko, 2016; Kahneman, 2003; Slovic et al., 2002). In the context of PA, the affect heuristic can be understood as an automatically evoked affective reaction to a given situation, most often shaped by past experiences, that may influence behavioral decisions (Ekkekakis and Zenko, 2016). As such, several authors have theorized that how one experiences the non-reflective form of afferent information constantly available to consciousness, called core-affect (Russell and Feldman Barrett, 1999), can, directly or indirectly, shape one’s exercise-related actions and choices through, at least in part, automatic paths (Brand and Cheval, 2019; Jekauc et al., 2024; Stevens et al., 2020).

This is one of the key assumptions of the Affective-Reflective Theory (ART) of physical inactivity and exercise (Brand and Ekkekakis, 2018), which aims to explore the psychological processes that govern momentary behavioral choices, particularly in relation to exercise. The ART is a dual-process theory and, as such, proposes the interplay of two systems: one fast, effortless, implicit, and affective (commonly referred to as System 1), and the other slower, deliberative, explicit, and reflective (commonly referred to as System 2). This framework emphasizes that human behavior and decision-making depend on the interactions of these two processes. Accordingly, automatic pathways – in which core affect plays a crucial role – are posited to influence behavior alongside reflective processes, jointly contributing to the final behavioral response (Simpson et al., 2025; Brand and Cheval, 2019; Zenko and Ekkekakis, 2019).

The degree of pleasure or displeasure that individuals experience during exercise appears to be associated with future exercise participation and PA behavior (Baldwin et al., 2016; Rhodes and Kates, 2015; Tavares et al., 2025). Therefore, the promotion of pleasure should be considered a key goal of exercise professionals (American College of Sports Medicine, 2025). Moreover, of the various factors that can influence the affective experience during exercise, intensity seems to play a particularly important role (Ekkekakis et al., 2011). As such, helping professionals in fitness centers understand how they can promote positive affective experiences through, for example, the modulation of exercise intensity could be beneficial for motivational purposes (Teixeira et al., 2022a). However, the exercise-science literature is virtually devoid of concrete recommendations and guidelines, as well as of experimental studies directly testing these assumptions, an absence that limits the accurate evaluation of the potential of this approach.

### Operationalizing theory-driven interventions: are we overcomplicating it?

Human behavior change is inherently complex and multifaceted, prompting the development of extensive analyses, frameworks, ontologies, and other conceptual resources to better understand it [e.g. (Knittle et al., 2020; Marques et al., 2024; Michie et al., 2011; Teixeira et al., 2020)]. However, the identification of several factors, such as implementation fidelity and intervention complexity, that may explain the limited impact of theoretically informed efforts to promote PA, has revealed the reverse side of this “coin.” Rather than enhancing practical outcomes, these elaborate approaches have, in some cases, undermined their own effectiveness and cast doubt on the real-world relevance of theory-based interventions (Rhodes et al., 2019; Simpson et al., 2025; Gourlan et al., 2016; Prestwich et al., 2014).

A recent critique of the difficulty in translating theoretical approaches into actionable outcomes within fitness contexts relates to the unrealistic expectation that exercise professionals are sufficiently knowledgeable and capable of interpreting and implementing such behavioral guidelines (Teixeira et al., 2024a). Fitness centers are highly dynamic, supervised exercise environments where professionals must manage a wide range of factors pertaining to their academic and professional training. Delving into the complementary, yet complex, domain of motivational science would require considerable prior preparation, ideally embedded in academic training. However, some reports have revealed limitations in the education of professionals and difficulty in applying even basic field-specific academic knowledge to practice (De Lyon and Cushion, 2013; Elder et al., 2003; Zenko and Ekkekakis, 2015). Moreover, as Teixeira et al. (2024a) noted, “for some exercise professionals, the gap between knowing some motivational theories/behavior change techniques and being able to apply them operationally may be a considerable shortcoming for their successful implementation” (p. 20). This would render, for example, the information contained in the behavioral techniques recommended in the ACSM guidelines (chapter 12) (American College of Sports Medicine, 2025) hard to translate into action. Given these challenges, assuming that complex and highly structured motivational approaches and behavior-change techniques are feasible in such contexts seems somewhat unrealistic.

Enriching the repertoire of exercise professionals’ interventions (e.g., evaluation, prescription, supervision, communication style) with theory-driven behavioral techniques has been proposed as a potential path toward improving the experience that participants derive from exercise programs and, by extension, exercise adherence (Teixeira et al., 2024a; Hancox et al., 2018; Teixeira et al., 2023). This would help circumvent excessively complex and impractical recommendations often emerging from the motivational and behavioral science research literature. The core idea is that introducing small, targeted modifications to habitual practices (e.g., instructional style, allowing some degree of autonomy in exercise selection, teaching intensity self-regulation, and taking individual preferences into consideration) would be easier for professionals to integrate into their routine. These changes would be sufficiently flexible to accommodate the diversity of activities and interactions that take place in fitness centers.

Two (of the very few) relevant examples of this approach, one representing each of the two theoretical perspectives employed in this study (i.e., SDT and hedonism), are the experimental approaches of Ntoumanis et al. (2017) and Teixeira et al. (2024b). In the former, quasi-experimental study, group fitness instructors participated in a training course designed to enhance their motivationally adaptive communication styles. This intervention was grounded in SDT principles, employing behavior-change techniques aimed at fostering interpersonal behaviors that support the basic psychological needs and the development of autonomous motivation [see also Hancox et al. (2015), for the study protocol]. While the course was, arguably, neither simple nor straightforward, the intended outcomes were relevant to practitioners, as the course focused on adapting everyday communication to encourage supportive motivational strategies and avoid maladaptive ones. The latter example, rooted in hedonic theory, involved a more direct and easily implementable intervention. This randomized controlled trial (Teixeira et al., 2024b) aimed to help exercisers experience pleasure during training by having professionals guide them in selecting exercise intensity based on individual preferences and affective responses, as well as by encouraging self-selected intensities [see (Teixeira et al., 2023), for the protocol]. In both cases, the interventions targeted typical professional practices, embedding practical motivational and behavioral strategies into everyday routines. From this perspective, the present study is not only an evaluation of two brief training programs, but also an empirical test of whether simplifying and operationalizing core behavioral theories can yield meaningful improvements in real-world exercise adherence, aligned with the most recent recommendations on the topic (Alsop et al., 2023; World Health Organization, Regional Office for Europe, 2022).

## Objectives

The present study aims to examine the associations of two short, behavioral theory–driven professional training programs: one based on SDT and the other grounded in hedonic principles. The primary outcome of interest is health-club attendance over one year. In addition, the study seeks to examine associations with these programs on individual behavioral and motivational outcomes among exercisers. The primary hypothesis is that fitness centers implementing either theory-driven training program will exhibit more favorable individual attendance trajectories over time compared with the control condition. Furthermore, it is hypothesized that the hedonic training approach will be associated with higher attendance than the SDT-based intervention. This expectation is based on (a) the relative ease and immediacy with which the hedonic framework and its context-specific tools and strategies can be applied (Teixeira et al., 2024b) and (b) the theoretical premise that SDT-based behavior change requires a longer period for integration and internalization (Ntoumanis et al., 2021). Secondary hypotheses include the following: (a) both theory-driven interventions will be associated with lower attrition rates than the control group and (b) the interventions will be associated with favorable changes over time in behavioral and motivational constructs, including exercise habit, intention to be physically active, satisfaction of basic psychological needs, behavioral regulation, enjoyment, and positive affective experiences.

## Methods

### Trial design

This will be a pragmatic, controlled intervention study with random allocation of three parallel training conditions (allocation ratio 1:1:1), using single-blinded outcome assessments. Intervention conditions are allocated at the fitness-center level, such that all exercise professionals within a given club are exposed to the same training condition. Although outcomes are measured at the individual member level, the primary unit of inference is the fitness center, with individual-level data used to characterize attendance trajectories and variability over time within each condition. The study will be conducted between September 2025 and August 2026. The training program will be applied in three health clubs belonging to the same company, to most of the exercise professionals employed at these clubs (> 80%), and at the beginning of the club season. Although all participating fitness centers belong to the same company, they operate with independent professional teams and geographically separated facilities. No systematic staff movement between the centers is expected during the intervention period. In addition, club managers were asked to avoid sharing the specific contents of the training sessions across centers throughout the implementation period. Training sessions were delivered independently within each fitness center according to the allocated condition.

Shorter content and operational/contextual strategies will be reinforced individually at approximately three-month intervals. For one year, all new club memberships and dropouts will be recorded. Moreover, approximately 39 exercisers per arm will be recruited to be monitored throughout the entire period, allowing the close assessment of changes in behavioral and motivational variables of interest. Considering that the clubs are business entities, the successful completion of the planned study will require close cooperation of the researchers with the managers of the fitness centers in planning the recruitment, advertising the study, and choosing the most appropriate time to intervene with each professional individually. The SPIRIT guidelines, as proposed by Chan et al. (2025), were followed for the development of the present protocol. The trial was preregistered on ClinicalTrials.gov with the ID number NCT07156240.

### Trial setting

The intervention will take place at three fitness centers in the broader metropolitan area of Lisbon, district of Portugal. All of them are classified as being in the mid-market price segment, medium-sized, and with similar characteristics (e.g., target clientele, services provided, number of staff members). They all belong to the same company but are geographically separated by a distance of at least 10 kilometers.

### Eligibility criteria

The eligibility criteria are structured according to the type of sample. First, regarding the exercise professionals receiving the intervention program, all will be eligible to receive the training course. At least 80% of the professionals who work full-time in the selected fitness centers must attend the initial training. For the remaining professionals, who may be absent from the initial training, an alternative session with the course contents and associated supporting materials will be provided within one week of the initial training date. Those who do not meet this condition (e.g., freelancers or those employed only as occasional substitutes) may, and are encouraged to, attend the course if interested.

The eligibility criteria for the exercisers will be structured as follows: for the primary outcome (i.e., attendance), (a) new members who enroll in the clubs after September 1st of 2025 during the intervention period will be eligible for intervention-specific analysis. Also, (b) all already-enrolled club members will be eligible for the global sample complementary analysis. Regarding the secondary outcomes (behavioral and motivational variables), individuals may voluntarily participate in the study if they are (i) over 18 years of age, (ii) new members or re-enrolled (after a minimum of six-month absence from any club) during September 2025, and (iii) until reaching double of the target sample size. No exclusionary criteria will be applied, as long as individuals are accepted as members of the clubs, which entails being classified as apparently healthy and/or without contraindications to exercise. No cross-contamination between clubs and exercisers is expected. The training course will be conducted by two of the researchers (unblinded to group allocation and the purposes of the study).

### Interventions

#### Training courses

All groups of exercise professionals will receive training under similar conditions and for similar durations. A three-hour initial course and workshop will be delivered, during which specific content will be presented for discussion and practical demonstration. Subsequently, four individually scheduled reinforcement sessions will be conducted throughout the year, approximately 10–12 weeks apart. During these sessions, the researchers will review the application of the content by each professional, identify challenges encountered during the previous period, and offer solutions through collaborative discussion. In addition, large-sized posters summarizing key messages from each training session will be displayed in the staff lounges following the initial training course. These posters will be updated during each of the first three individual reinforcement sessions. Although the content and training are expected to influence future interactions with clients, professionals will be under no obligation to alter their usual practice, as the content of the training and associated materials will be offered as optional, to be used and applied at their discretion, and depending on the specific needs of individual clients.

For the control group (Control), the training course will focus on reviewing and updating content related to the Frequency, Intensity, Time, and Type principle (FITT) (American College of Sports Medicine, 2025). The first part of the initial training will be educational, while the second part will involve a discussion of case studies and methods for implementing guideline-recommended procedures. No specific content regarding the promotion of exercise adherence will be included beyond general considerations typically associated with the FITT principle.

The SDT-based course (Experimental Group I) will include: (i) an introduction/review of the basic psychological needs and organismic integration subtheories; (ii) strategies for supporting the development of psychological needs; (iii) a motivational assessment instrument (behavioral regulation, in a simplified version); and (iv) discussion of case studies and practical applications. The selected content aims to foster understanding of core concepts, including the importance of satisfying BPN to promote behavioral internalization and integration, as well as the role of autonomous (vs. controlled) motivation in enhancing well-being and adherence. A detailed explanation of basic needs and how to operationally support them (in various gym-related contexts) will be provided. In addition, information on how to identify the tendency of individual clients for behavioral regulation during regular interactions, drawing on, but not directly echoing, several examples from the exercise-science and SDT literatures (Ryan and Deci, 2017; Teixeira et al., 2020; Hancox et al., 2018), will also be provided. A simplified, one-item-per-construct version of the Behavioral Regulation In Exercise Questionnaire 4 (BREQ-4) for motivational quality assessment, based on suggestions by Rocchi et al. (2023), will be introduced, both to facilitate conceptual understanding and to add to the toolset of the exercise professionals for future reference. Case studies simulating real-world scenarios of interactions with clients will be used to facilitate the integration of this knowledge into the daily practice of exercise professionals. Specific examples of the intervention can be found in the Supplementary file 1a (SDT-based communication adaptations) and Supplementary file 1b (single-item proposal for behavioral regulation).

The hedonic-based course (Experimental Group II) will focus on: (i) introducing or revisiting foundational postulates of hedonic theory and related approaches (e.g., affect heuristic, affective-reflective theory); (ii) presenting related techniques for operationalizing these postulates (e.g., self-regulation of exercise intensity, structuring intensity “peaks” during sessions, and adjusting cool-downs to enhance end-of-session affect) aimed at enhancing affective responses, structured in three main domains: (a) professionals’ intensity adjustments aiming positive affective responses, (b) exercisers’ intensity self-selection guidance towards pleasurable sensations, and (c) professionals’ promotion of a pleasure seeking, displeasure-avoiding session experience; (iii) familiarization with the use of self-report instruments for assessing affective valence [Feeling Scale (FS) (Brito et al., 2022)], one item simplified versions of intensity preference and tolerance and respective agreement (PRETIE-Q) (Teixeira et al., 2021) and enjoyment (PACES) (Bastos et al., 2025b), and affective expectations [adapted empirical valence scale (EVS) (Teixeira et al., 2024b; Lishner et al., 2008)], thus facilitating individual tailoring of the sessions; and (iv) case study discussions and implementation strategies. This structure is informed by a range of studies and recommendations related to session organization (Bastos et al., 2025a; Ekkekakis et al., 2023), the impact of exercise intensity on affective responses (Ekkekakis et al., 2011; Teixeira et al., 2022a), contextual implementation strategies (Evmenenko and Teixeira, 2020; Williams and Rhodes, 2023; Santos and Teixeira, 2023), and evidence linking pleasure to increased exercise frequency (Tavares et al., 2025; Teixeira et al., 2024b). More information about the content of this intervention can be found in the Supplementary file 2a (hedonic-based communication adaptations) and Supplementary file 2b (single-item proposal).

No major operational barriers to implementation are anticipated, as fitness centers will continue their regular operations throughout the intervention period. Prior coordination with club managers will aim to ensure at least 80% attendance of the training sessions among full-time professionals, as well as the use of internal communication channels to distribute training materials to those unable to attend. An additional course training session may be offered within the same month if unforeseen circumstances prevent meeting the minimum attendance rate of 80%. Client enrollment in each group will be supported by the public relations office of the clubs, the front desk, and the marketing departments. No other strategies to enhance adherence/attendance will be introduced during the intervention period, to prevent bias in the outcome variables (i.e., club attendance and dropout). Because the study was designed to preserve the naturalistic characteristics of routine professional practice, variability in the application of the proposed strategies across professionals and centers is expected. Professionals hired throughout the year will be offered training sessions, contingent on their willingness to participate. Participants may withdraw from the study at any point without consequences. Requests for data removal will be honored whenever identifiable data are still available and traceable. Data that have already been anonymized and incorporated into aggregated analyses may no longer be removable, in accordance with GDPR and institutional data-protection procedures.

### Outcomes

The primary outcome variable will be club attendance, which, for the designated fitness centers, will only reflect club visits with the intent to exercise and not access complementary services (i.e., spa, well-being centers, coffee lounges). This will be assessed across all trimesters, with particular emphasis on one-year results (as the primary outcome endpoint). Comparisons will be made over time within and across intervention conditions for both new members enrolling between September 1, 2025, and August 31, 2026, and members already enrolled prior to this period, for whom the intervention may reflect a change from their prior club experience. The secondary outcomes will be behavioral (attrition rate, namely the number of membership cancellations or dropouts as a percentage of the total number of new members in a given time period; see Methods section for details) and motivational constructs (exercise habit, intention to be physically active, satisfaction of basic psychological needs, behavioral regulation, enjoyment, and affective exercise experiences), in line with the study’s secondary hypotheses regarding potential motivational and behavioral changes associated with the intervention conditions. These will be assessed in a subsample of participants who will voluntarily take part in the longitudinal evaluations. Additional variables (i.e., state self-control, intensity agreement, course content evaluation, one-year pre-intervention attendance, and attrition patterns at the club level, and the degree and frequency of application of the knowledge provided during the training course) will be included for descriptive or exploratory tertiary analyses. No harm is expected from the intervention.

### Participant timeline

The study is expected to run between September 2025 and the end of August 2026. The three health clubs will be randomized to the conditions (i.e., control, experimental 1 [SDT-based course], and experimental 2 [hedonic-based course]) at the beginning of this period. Baseline motivation and behavior assessments will be conducted upon enrollment after verification of the inclusion and exclusion criteria. These variables will be assessed again at six and 12 months post-randomization. The training courses will be held at the beginning of September at each club according to the conditions previously randomized. Thereafter, short, individualized discussions with each of the exercise professionals, focusing on reinforcing the ideas presented at the initial training courses and providing support for implementation, will take place approximately 10 to 12 weeks apart until the end of August 2026. The application of the content knowledge delivered during the respective training courses (i.e., frequency and fidelity of implementation) will be assessed during each visit. Attendance and dropout data will be collected continuously. Table 1 presents the participant timeline and sequencing of recruitment, allocation, intervention, and assessments.

**Table 1:** Participants’ enrollment, allocation, post-allocation, and close-out timeline.

	Study period						
	Enrollment	Allocation	Post-allocation				Close-out
Timepoint	-t1	0	t1	t2	t3	t4	t5
Enrollment							
Eligibility screen	⨯						
Informed consent	⨯						
Allocation (of the clubs)		⨯					
Interventions							
Control			⨯	⨯	⨯	⨯	
Experimental (I)—SDT			⨯	⨯	⨯	⨯	
Experimental (II)—hedonic			⨯	⨯	⨯	⨯	
Assessments							
Club’s members							
New memberships			⨯	⨯	⨯	⨯	⨯
Dropouts			⨯	⨯	⨯	⨯	⨯
Exercise frequency			⨯	⨯	⨯	⨯	⨯
Exerciser’s sample							
Sociodemographic	⨯						
Behavioral regulations	⨯				⨯		⨯
Basic psychological needs	⨯				⨯		⨯
Exercise enjoyment	⨯				⨯		⨯
Attraction-antipathy	⨯				⨯		⨯
Pleasure-displeasure	⨯				⨯		⨯
Preference/Tolerance	⨯				⨯*		⨯*
Exercise habit	⨯				⨯		⨯
Intention to be physically active	⨯				⨯		⨯
State self-control	⨯				⨯		⨯
Professionals							
Course comprehension test			⨯				
Course knowledge application				⨯	⨯	⨯	⨯

t1 = Baseline assessments; t2 = 3 months; t3 = 6 months; t4 = 9 months; t5 = 12 months; * = only agreement items.

### Sample size

Sample size determination follows SPIRIT (Chan et al., 2025) guidance for pragmatic interventions in which traditional power calculations are constrained by design features. As such, considerations were made while considering the nature of the intervention, the fitness-center–level allocation of training conditions, and the longitudinal structure of the primary outcome. Given that only three centers are included, formal power calculations for between-condition hypothesis testing based on individual-level randomization are not appropriate. Instead, sample size adequacy is primarily related to the large number of individual observations and repeated measurements, which support the longitudinal estimation of attendance trajectories over time within the constraints of the present pragmatic design.

For the primary outcome (attendance), all eligible members of the participating fitness centers will be included in the analyses at three, six, nine, and 12 months, although the focus will be given to those enrolled after September 1, 2025. Each participating fitness center has more than 2,000 enrolled members at any given time, and historical records indicate that approximately 200 new members typically enroll in each club during the month of September. This provides a high-density longitudinal dataset, with multiple repeated observations per individual and sufficient sample size in each intervention condition to support longitudinal mixed-effects modeling of attendance trajectories.

As for the secondary outcomes, these will focus on (i) dropout rates at three-month intervals up until one year, and (ii) motivational variables. In the case of dropout rates, administrative records from all members enrolling after September 1, 2025, will be used. This outcome is derived from the complete population of new members during the intervention period, and the expected number of observed events over the 12-month follow-up is sufficient to support descriptive and time-to-event analyses of attrition patterns across intervention conditions. In the case of the motivational variables, the most restrictive analyses will be conducted in a subsample of exercisers enrolling in September. These participants will be recruited for the longitudinal assessment of behavioral regulation, satisfaction of basic psychological needs, exercise enjoyment, subjective exercise experience, exercise habit, and intention to be physically active, assessed at baseline, six months, and twelve months. Sample size considerations for this subsample were guided by the most restrictive planned within-participant longitudinal comparisons and estimated using G*Power (v.3.1.9.7) (Faul et al., 2009). Using benchmarks from a related work [e.g. (Teixeira et al., 2023; Andrade et al., 2022; Henriques et al., 2023)], a repeated-measures ANOVA assuming a small-to-moderate magnitude of change over time (*f* = 0.20), an *α* level of 0.05, statistical power (1-*β*) of 0.95, three repeated measurements, a correlation between repeated measurements of approximately r = 0.70, and allowing for potential violations of sphericity (*ε* ≈ 0.70), a minimum of approximately 53 participants completing follow-up assessments across the three fitness centers is expected to provide adequate sensitivity to characterize meaningful longitudinal changes in motivational outcomes over time. Linear mixed-effects models will be used for analysis, as they are expected to be at least as efficient as the repeated-measures assumptions used for these calculations and allow the inclusion of all available data in the presence of missing observations. Given that the average attrition rate in Portugal over a one-year period in these contexts has been reported as ranging between 50 and 60% (Pedragosa and Ferreira, 2024), a total sample of approximately 117 participants (≈55% attrition) will be required.

### Recruitment, allocation, and blinding

The health clubs were selected due to their standardized business practices across all clubs (thus reducing potential bias associated with heterogeneity). Also, all clubs have an electronic tracking system that allows the monitoring of attendance, specifically in the designated exercise areas. The control and experimental groups will be allocated through a simple randomization procedure performed by the primary investigator using the tool provided by randomizer.org. Because all centers were allocated simultaneously prior to intervention implementation, no separate allocation concealment procedure was performed. Newly enrolled members will be recruited on a voluntary basis in all clubs through information that will be provided at the reception desks and by the sales teams at the time of registration. The recruitment of new members will occur only during the month of September on a rolling basis. A personal training session will be offered to all those who agree to participate in the three assessments of the study. Exercise professionals were aware that they were participating in a study involving professional training and follow-up reinforcement sessions related to exercise promotion and communication strategies. However, they were not informed of the specific study hypotheses linking the training contents to longitudinal attendance, attrition, and motivational outcomes among exercisers. From the professionals’ perspective, the intervention was presented primarily as a professional-development opportunity intended to support evidence-informed exercise-promotion practices. Exercisers participating in the longitudinal assessments were unaware of intervention allocation and of the specific hypotheses being examined. They were informed only that the study aimed to assess the longitudinal development of motivational and behavioral variables associated with exercise participation. Due to their role in intervention delivery and study coordination, the research team could not be considered blinded to intervention allocation. However, the primary attendance and dropout outcomes were obtained from administrative fitness-center records, thereby reducing the potential influence of participant expectancy effects on the primary outcome measures. Nevertheless, because exercise professionals were aware of the training content they received, the possibility of performance-related influences on professional behavior cannot be fully excluded and will be considered when interpreting future outcome findings.

### Data collection methods

#### Participants (fitness club members)

##### Basic psychological needs

The Portuguese version of the Basic Psychological Need Satisfaction and Frustration Scale in Exercise (BPNSFS-E) (Rodrigues et al., 2019) will be used. This instrument includes 24 items, 12 assessing need satisfaction and 12 assessing need frustration, which are theoretically aligned with constructs from SDT (Vansteenkiste et al., 2020). The scale evaluates satisfaction of autonomy (e.g., “I have a feeling of freedom and choice in the things I make”), competence (e.g., “I feel confident that I can do things right”), and relatedness (e.g., “I feel that the people I care for also care for me”), as well as frustration of autonomy (e.g., “I feel the majority of the things I do are out of obligation”), competence (e.g., “I feel insecure about my abilities”), and relatedness (e.g., “I feel excluded from the group I want to belong to”). Responses are provided on a 5-point Likert scale ranging from 1 (“Totally disagree”) to 5 (“Totally agree”).

##### Behavioral regulations

The Portuguese version of the Behavioral Regulation in Exercise Questionnaire (BREQ-4) (Teixeira et al., 2022b) will be used to assess behavioral regulation according to the organismic integration sub-theory of SDT (Ryan and Deci, 2017). This instrument includes 28 items covering all behavioral regulations, including the dual facets of introjected regulation: (e.g., amotivation: “I do not see why I should have to exercise”; external regulation: “I exercise because other people say I should”; introjected avoidance regulation: “I feel guilty when I do not exercise”; introjected approach regulation: “I feel proud of myself when I persist”; identified regulation: “I value the benefits of exercise”; integrated regulation: “I exercise because it is consistent with life goals”; and intrinsic motivation: “I exercise because it’s fun.”) Responses are provided on a 5-point Likert scale ranging from 0 (“Not true for me”) to 4 (“Very true for me”). The 21-item version that demonstrated the best model fit in this context will be employed (Teixeira et al., 2022b).

##### Exercise enjoyment

The Portuguese version of the Physical Activity Enjoyment Scale (PACES) (Bastos et al., 2025b) will be used to assess enjoyment of exercise. This instrument contains 18 items using a 7-point bipolar scale (e.g., “It’s no fun at all – It’s a lot of fun”).

##### Affective exercise experiences

The attraction-antipathy and the pleasure-displeasure scales of the Affective Exercise Experiences Questionnaire (AFFEXX) (Ekkekakis et al., 2021) will be used. The first scale is a motivational outcome variable consisting of five items assessing the tendency to approach or avoid exercising (e.g., “Exercise is low on my priority list—Exercise is high on my priority list”); the second, is a core affective experience assessment, evaluated with four items (e.g., “Exercise makes me feel worse—Exercise makes me feel better”). Both are answered on a 7-point bipolar scale. A translation and back-translation of the full questionnaire was performed following several recommendations for adequate instrument translation and adaptation (Brislin, 1970; Brislin, 1980) and has been used in the context of exercise in fitness clubs [e.g., (Teixeira et al., 2023)].

##### Intensity preference/tolerance

Preference for and tolerance of exercise intensity, and their agreement with individually experienced intensity, will be assessed using the Portuguese version of the Preference for and Tolerance of Exercise Intensity Questionnaire (PRETIE-Q) (Teixeira et al., 2021). The instrument is composed of 10 items (five for each factor; preference: “While exercising, I prefer activities that are slow-paced and do not require much exertion”; tolerance: “I always push through muscle soreness and fatigue when working out”) answered on a 5-point Likert scale ranging from 1 (“Totally disagree”) to 5 (“Totally agree”). The two additional items included in this version of the instrument to assess exercise intensity preference (“The intensity of my workout is in agreement with my preference”) and tolerance (“The intensity of my workout is in agreement with my tolerance”) will also be used to evaluate participants’ perceptions of the prescribed workout intensity relative to their individual preferences.

##### Exercise habit

The Portuguese version of the Self-Report Behavioral Automaticity Index (SRBAI) (Rodrigues et al., 2021) will be used to measure exercise-related habit formation. This scale contains four items (e.g., “I do [it] without thinking”), answered on a 7-point Likert scale ranging from 1 (“Totally disagree”) to 7 (“Totally agree”).

##### Intention to be physically active

Behavioral intention will be measured using a single item: *“I will continue to practice physical exercise in the next 6 months as I recently practiced or in a very similar way (same type, frequency, duration, and intensity),”* based on previous studies in the Portuguese context (Teixeira et al., 2021) and grounded in Ajzen’s (2006) theoretical work. Responses are given on a 7-point bipolar scale ranging from 1 (“Absolutely not”) to 7 (“Absolutely yes”).

##### State self-control

The Portuguese version of the Brief Self-Control Scale (BSCS) (Pechorro et al., 2018) will be used to assess trait self-control. The instrument includes 13 items (e.g., “It is hard for me to break bad habits”), reflecting a unidimensional construct. Responses are given on a 5-point Likert scale ranging from 1 (“Totally false”) to 5 (“Totally true”).

##### Club attendance and attrition rate

Attendance data will be obtained through the electronic system that tracks the entrances and exits from the turnstile systems of the clubs. These data will be collected for the entire season (September – August) for all members, when the following condition is present: (a) active payment + (b) a minimum of 1 attendance per month^1^. The attrition rate will be calculated for each 3-month period and the entire season with the following formula: Attrition Rate = (Number of Cancellations or Dropouts / Total Number of Members) × 100. New club members who enrolled in the fitness clubs after September 1, 2025, will also be identified separately for subgroup analysis.

#### Exercise professionals

##### Course comprehension test

An anonymous course comprehension test will be administered immediately following course completion as a manipulation check. The test will include 10 multiple-choice questions with five response options each (e.g., “When a client says they decided to train in order to ‘lose belly fat,’ the most evident type of motivational regulation is: (a) external regulation, (b) introjected regulation, (c) identified regulation, (d) integrated regulation, or (e) intrinsic motivation” and “Integrating hedonic principles into exercise supervision and prescription can be as simple as: (a) aligning training intensity with what is individually preferred and tolerated, (b) adjusting speed/cadence/load in order to experience pleasurable sensations, (c) selecting activities/exercises/classes that are individually perceived as fun and enjoyable, (d) all of the above are correct, or (e) all of the above are incorrect”). Items are designed not only to assess recall but also to evaluate the ability to integrate and apply course content. Scores will be expressed as a percentage (0–100%), with incorrect answers penalized by deducting 1/5 of the value of each question.

##### Course-knowledge application test

During the four scheduled individualized sessions with professionals (T2 to T5; experimental groups only), participants will respond to two questions assessing the application of course content. These will be answered on 5-point Likert scales focused on (a) frequency (“How often do you use the knowledge or tools/strategies conveyed in the training course at the beginning of the season? 1 – Never; 5 – Always”), and (b) the degree (“To what extent do you translate the course content to your professional practice?; 1 – Superficially; 5 – Extensively”) of content application.

### Data management

Data will be collected and entered by the two researchers responsible for delivering the training courses. The final dataset will be anonymized and securely stored on a private server, in compliance with the General Data Protection Regulation (GDPR) of the European Union. Regular bimonthly checks will be conducted to ensure data quality and integrity. Access to the anonymized dataset can be requested from the primary investigator and will be considered upon review.

### Statistical methods

Preliminary analyses will be conducted prior to all statistical procedures to examine data quality, missingness patterns, and identify potential violations of statistical assumptions. All analyses are planned to be performed using SPSS v.27.0 (IBM, Co.). The primary outcome of the study is attendance, which will be analyzed both for all fitness center members and separately for those who joined the clubs after the commencement of the study (i.e., after September 1, 2025). Attendance will be operationalized as the individual’s mean weekly attendance within each three-month interval and assessed at four time points during the 12 months of the study (three, six, nine, and twelve months). Primary analyses of attendance will be conducted using linear mixed-effects models (LMMs), with time specified as a within-subject factor and individuals treated as random effects to account for repeated observations. Intervention condition (control, SDT-based training, and hedonic-based training), time, and their interaction will be included as fixed effects, with age and sex included as covariates. These models will be used to characterize and descriptively compare attendance trajectories over time across intervention conditions, with emphasis on the magnitude and direction of longitudinal patterns rather than on individual-level causal inference. Primary analyses will follow an intention-to-treat principle adapted to the cluster-allocated design, such that all eligible individuals are analyzed according to their fitness center’s assigned condition, regardless of attendance patterns or dropout. Linear mixed-effects models were selected because they incorporate all available longitudinal observations under a missing-at-random assumption without requiring imputation.

Because intervention allocation occurs at the fitness-center level and each condition is implemented in a single center, cluster-level variance and intraclass correlation coefficients cannot be estimated independently of condition-related effects. Accordingly, comparisons across intervention conditions are considered exploratory and descriptive in nature. For complementary descriptive purposes, 3 (Condition) × 4 (Time Points) split-plot ANOVAs may be conducted to summarize overall patterns of longitudinal change in attendance. Results from these analyses will be interpreted descriptively, given that intervention conditions are implemented at the fitness-center level.

Secondary outcomes include dropout and motivational variables. Dropout (membership cancellation) will be examined using administrative records from all members enrolling after September 1, 2025. Dropout rates will be summarized at three-month intervals, and time-to-event analyses will be used to describe adherence patterns over the 12-month period. Percentage attrition rates will be reported descriptively, and Kaplan–Meier survival curves will be used to illustrate the timing of dropout across conditions. Participants who remain actively enrolled at the end of the observation period will be treated as censored observations at their last available follow-up time point. Motivational and behavioral variables will be analyzed in the longitudinal subsample of participants. Changes over time in these variables (baseline, six, and twelve months) will be examined using linear mixed-effects models, with time specified as a within-subject factor and participants treated as random effects. Intervention condition, time, and their interaction will be included as fixed effects, with age and sex entered as covariates. Given the limited number of repeated measurements, models will be specified with a parsimonious random-effects structure. For consistency with prior literature and ease of interpretation, complementary 3 (Condition) × 3 (Time Points) split-plot ANOVAs may also be conducted, with results interpreted descriptively. Effect sizes will be reported as model-based estimates of change over time (e.g., differences in attendance slopes and estimated marginal means), with 95% confidence intervals. Additional control, complementary, and exploratory analyses are anticipated, particularly within ancillary studies.

### Ethics and dissemination

The present trial is registered under the ID number NCT07156240. Ethics approval was obtained from the institutional ethics committee of the university of the primary investigator, prior to the commencement of the study. All procedures will be conducted in accordance with international ethical standards for research involving human participants (World Medical Association, 2013). Club manager authorization and signed informed consent will be obtained from professionals and participants during the recruitment phase by two of the researchers. All will retain the right to withdraw from the study at any point without any negative consequences. Study enrollment and participation will be suspended if any major adverse events are identified, following international guidelines (Medical Research Council, 2017), although, given the nature of the study, no adverse events are expected. Should protocol amendments become necessary, additional approval will be sought from the institutional ethics committee, and participants and clubs will be informed of any changes and their potential implications. The leading author will periodically monitor the trial development.

The results of this trial will be published in peer-reviewed scientific journals. Preliminary, intermediate, or secondary findings may also be presented at national or international scientific conferences. Baseline data will not be analyzed until the intervention period has concluded. The final, coded, and anonymized dataset will be managed by the primary investigator (DT). Authorship will be determined in accordance with the criteria established by the International Committee of Medical Journal Editors.

## Data Availability

The raw data supporting the conclusions of this article will be made available by the authors, without undue reservation.

## Footnotes

1 Dropout will be considered when one of these conditions is not present. An exception will be created for those with active payment and only 1 month of absence in the 12-months period. This exception criterion was introduced to avoid misclassifying temporary absences as dropout.
