# Supplementary material for: Impact of two brief behavioral theory–driven professional training programs on fitness center attendance: protocol for pragmatic controlled intervention with random allocation
Source: Front Psychol. 2026 Jul 2;17:1856891. doi: 10.3389/fpsyg.2026.1856891 (PMC13372327; doi:10.3389/fpsyg.2026.1856891)
Supplement: Supplementary file 2 [file Supplementary_file_2.docx]

WHO Trial Registration Data Set

| Data category | Information |
| --- | --- |
| Primary registry and trial identifying number | ClinicalTrails.gov; ID: NCT07156240 |
| Date of registration in primary registry | 27/08/2025 |
| Secondary identifying numbers | None |
| Source(s) of monetary or material support | None |
| Primary sponsor | None |
| Secondary sponsor(s) | None |
| Contact for public queries | diogo.teixeira@ulusofona.pt |
| Contact for scientific queries | diogo.teixeira@ulusofona.pt |
| Public title | Impact of two brief behavioral theory–driven professional training programs on fitness center attendance: protocol for pragmatic controlled intervention with random allocation |
| Scientific title | Impact of two brief behavioral theory–driven professional training programs on fitness center attendance: protocol for pragmatic controlled intervention with random allocation |
| Countries of recruitment | Portugal |
| Health condition(s) or problem(s) studied | Behavioral modification towards exercise attendance and adherence |
| Intervention(s) | Control group: ACSM-FITT training course; experimental group 1: Self-determination theory operational content delivery; experimental 2: hedonic-based operational content delivery |
| Key inclusion and exclusion criteria | Adults, both sexes, enrolled in fitness center activities |
| Study type | Pragmatic, single-blind, controlled intervention study with three parallel arms |
| Date of first enrollment | September 1, 2025 |
| Target sample size | Three clubs; 6000 exercisers; 114 behaviorally accompanied exercisers |
| Recruitment status | Completed |
| Primary outcome(s) | Exercise attendance |
| Key secondary outcomes | Dropout, motivational status |
