# Supplementary material for: Impact of two brief behavioral theory–driven professional training programs on fitness center attendance: protocol for pragmatic controlled intervention with random allocation
Source: Front Psychol. 2026 Jul 2;17:1856891. doi: 10.3389/fpsyg.2026.1856891 (PMC13372327; doi:10.3389/fpsyg.2026.1856891)
Supplement: Supplementary file 3 [file Table_1.docx]

Supplementary file 1a - Examples of SDT-based communication/behavior adaptations provided in the professional’s course content

| Domain of action | | Interpersonal communication and behavior | BPN fulfillment rationale |
| --- | --- | --- | --- |
| During individualized sessions (e.g., personal training) or interactions | Autonomy | "Now we need to do some leg exercises to strengthen your quadriceps. Would you prefer doing the leg press or a squat?" | Providing choice |
|  |  | "I think it would be beneficial to perform a 20-minute cardiovascular workout in this session. Would you prefer to do it continuously or in two 10-min parts?" | Providing choice |
|  |  | "For your workout goals, we should increase your exercise weekly freq. or your daily levels of PA to increase energy expenditure. and reduce sedentary behavior. Which do you feel is more easily achievable?" | Presenting a rationale/explanation |
|  | Competence | "I think it is best to make a small correction to this exercise to improve technique. Let's adjust it to a different version." | Exercise regression to increase success in task; optimal challenge |
|  |  | "I see now that you are ready to step up your game. Let's increase the difficulty of this exercise/session!" | Providing optimal challenge |
|  |  | "What a great workout today. You have really improved during the last weeks!" | Providing positive performance feedback |
|  | Relatedness | "I feel that you are struggling with this workout. It is indeed a bit challenging, but we can adjust it if we need to." | Providing security and concern |
|  |  | "Do you feel tired today? We can ease the workout a little bit if needed." | Providing security and concern |
|  |  | "I know that [insert exerciser objective] is hard, but I will try to help you achieve your goals." | Showing respect and caring for one's difficulties |
| General prescription or PA-oriented counseling | Autonomy | "It would be important to your goal to perform 20-min of cardiovascular exercises. Between the treadmill, the bicycle, and the stepper, which one do you prefer?" | Providing choice |
|  |  | "In your next workout plan, would you like to keep the same stretching exercises, or do you prefer to change the exercises/dynamics a little bit?" | Providing choice |
|  |  | "This exercise is important for you because of [insert rational]. We can adjust or modify it if needed, but let's try to keep it in your sessions." | Presenting a rationale/explanation |
|  | Competence | "That exercise seems very uncomfortable. Can I help you adjust it to improve technique?" | Providing feedback |
|  |  | "I noticed that your treadmill running pace and inclination were very demanding at this stage. Let's adjust it slightly." | Providing feedback |
|  |  | "Your technique in these exercises is now good. Let's advance to new ones!" | Providing positive performance feedback; providing optimal challenge |
|  | Relatedness | "I noticed that you haven't come to the gym lately. Is there anything I can help you with related to your workout?" | Showing respect and caring for one's difficulties |
|  |  | "How did you feel after your last workout? We can adjust some aspects to reduce some soreness or fatigue." | Showing respect and caring for one's difficulties |
|  |  | "Whenever you feel that you are struggling with an exercise or have any doubt, call me. I am here to help you!". | Supporting an inclusive environment |
| Group classes or activities | Autonomy | "Right now we have two classes starting, one more adjusted to increase cardiovascular resistance, and another to improve flexibility. Which would you like to experiment?" | Presenting a rationale/explanation |
|  |  | "The next circuit will have a 30s duration per exercise. If you feel it is too long, use the last 5s to rest and prepare for the next exercise!" | Providing choice |
|  |  | "In the next set, we will use a bar and some weights. Go ahead and select two weights between 2,5kg and 10kg according to your energy today." | Providing choice |
|  | Competence | "Let's take it up a notch and try the [insert exercise variant] next time." | Providing optimal challenge |
|  |  | "What a great session! Your rhythm is now perfect!" | Providing positive performance feedback |
|  |  | "For those struggling with this exercise, remember to use your arms to help you jump higher!" | Providing feedback |
|  | Relatedness | "Everybody, this is [insert exerciser's name]. Let's make him/her feel welcomed in our class!" | Supporting an inclusive environment |
|  |  | "I noticed you are still struggling with the choreography. It is perfectly normal. It takes some time to remember all the steps." | Showing respect and caring for one's difficulties |
|  |  | "Next, we will do a new routine. Don't worry if you don't pick it up right away. It is hard to do these new moves." | Showing respect and caring for one's difficulties |
