# Supplementary material for: Impact of two brief behavioral theory–driven professional training programs on fitness center attendance: protocol for pragmatic controlled intervention with random allocation
Source: Front Psychol. 2026 Jul 2;17:1856891. doi: 10.3389/fpsyg.2026.1856891 (PMC13372327; doi:10.3389/fpsyg.2026.1856891)
Supplement: Supplementary file 4 [file Table_2.docx]

Supplementary file 1b - Single items for professionals' behavior regulation assessment/identification (BREQ-4) and key points examples to identify the motivational quality

| Behavioral regulations | | Single items per construct | Key points to identify during interactions with exercisers | |  |
| --- | --- | --- | --- | --- | --- |
|  | Amotivation | I can’t see why I should bother exercising (item 8) | The exerciser says things like: | "I don't really know why I keep working out."; I come to the gym just because I am paying the monthly fee." | |
| Controlled | External regulation | I exercise because others will not be pleased with me if I don’t (item 16) |  | "My physician/friend/partner says I must lose some weight"; "I need to lose some weight for the summer season"; "Today is pizza night. I need to burn some extra calories for later!"; "I want to show them I can get huge (muscularity)!" | |
|  | Introjected (avoidance) regulation | I feel guilty when I don’t exercise (item 3) |  |  |  |
|  | Introjected (approach) regulation | Because I want to prove to myself that I’m able to persist (item 11) |  |  |  |
| Autonomous | Identified regulation | It’s important to me to exercise regularly (item 12) |  | "I have been exercising for as long as I can remember."; I like coming to the gym to alleviate stress."; "I want to have energy and health for my retirement age."; "I try to prioritize my health."; I prefer the XWZ activity because it's dynamic and fun." | |
|  | Integrated regulation | I consider exercise to be part of my identity (item 13) |  |  |  |
|  | Intrinsic motivation | I enjoy my exercise sessions (item 14) |  |  |  |
