# Supplementary material for: Impact of two brief behavioral theory–driven professional training programs on fitness center attendance: protocol for pragmatic controlled intervention with random allocation
Source: Front Psychol. 2026 Jul 2;17:1856891. doi: 10.3389/fpsyg.2026.1856891 (PMC13372327; doi:10.3389/fpsyg.2026.1856891)
Supplement: Supplementary file 5 [file Table_3.docx]

Supplementary file 2a - Examples of hedonic-based communication/behavior adaptations provided in the professional’s course content

| Domain of action | Interpersonal communication and behavior | Rational | | |
| --- | --- | --- | --- | --- |
| Professionals' intensity adjustments aiming for positive affective responses | When prescribing and supervising, prioritize: | A | A1 | Aligning exercise intensity to the one's individually preferred and tolerated |
|  |  |  | A2 | Using the FS to assess the affective response and promote an intensity adjustment towards pleasurable sensations |
|  |  |  | A3 | Asking how one is feeling during a class/session and interpreting bodily feelings expressed as proxies of the affective response |
|  | | | | |
| Exercisers' intensity self-selection guidance towards pleasurable sensations | Educate and advise on: | B | B1 | Regulating the speed/cadence/load to experience pleasurable sensations throughout the workouts |
|  |  |  | B2 | Continuously interpreting bodily signs of displeasure or low pleasure and regulating practice to improve them |
|  |  |  | B3 | Incentive autonomous decisions towards exercise intensity adjustments, aiming for pleasurable sensations |
|  | | | | |
| Professionals' promotion of a pleasure-seeking, displeasure-avoiding session experience | Promote as possible: | C | C1 | Selection/advise of individually perceived fun or enjoyable activities/exercises/routines |
|  |  |  | C2 | Identification of the potentially unpleasant moments of the session/routine (planning) and adjust them to ensure a positive affective response |
|  |  |  | C3 | Ending the session (final minutes) on an affective "high note" |
|  |  |  | C4 | Inquire about the expected pleasure/enjoyment of the upcoming session after a brief explanation of what is expected to be performed |

|  | Operational examples | | | | | | | | | | | | | | | | |  |
| --- | --- | --- | --- | --- | --- | --- | --- | --- | --- | --- | --- | --- | --- | --- | --- | --- | --- | --- |
| A1 | Low score on preference AND tolerance (e.g., < 3) would suggest selecting lower intensities and fewer intensities changes (e.g., continuous aerobic activities, lower RM%; higher scores on preference AND tolerance allow the exploration of higher intensities and manipulations (e.g., interval training; higher RM%); low preference AND high tolerance tends to favor longer workouts; and high preference AND low tolerance tends to favor shorter workouts but with some (not exclusively) exposition to higher intensities; search for intensity preference and tolerance agreement scores > 3 | | | | | | | | | | | | | | | | |  |
|  |  |  |  |  |  |  |  |  |  |  |  |  |  |  |  |  |  |  |
| A2 | FS scores < 0 need immediate attention to the experienced intensity; FS scores between 0 and 2 are suggestive of intensity adjustments; FS scores > 2 are suggestive of an optimal experience | | | | | | | | | | | | | | | | |  |
|  |  |  |  |  |  |  |  |  |  |  |  |  |  |  |  |  |  |  |
| A3 | Expressions like: "I am feeling very tired"; "This is very hard"; "I feel exhausted", "This is painful." | | | | | | | > | All are suggestive of an excessive exercise intensity (and displeasure) and may require adjustments | | | | | | | | |  |
|  | Behaviors like: Grasping for air, extreme panting, falling to the ground to rest | | | | | | |  |  |  |  |  |  |  |  |  |  |  |
|  |  |  |  |  |  |  |  |  |  |  |  |  |  |  |  |  |  |  |
| B1 | "A 20-min run doesn't need to be performed always at 12 km/h. Instead, allow speed adjustments to account for pleasure-displeasure variations."; "When you feel that your day was exhausting, try to adjust the load slightly down to experience a more pleasurable workout." | | | | | | | | | | | | | | | | |  |
|  |  |  |  |  |  |  |  |  |  |  |  |  |  |  |  |  |  |  |
| B2 | "Exercise may make you feel bad or uncomfortable, and we should avoid it. Instead, talk to me or make small adjustments in intensity whenever you feel lacking energy, muscle pain, or increased fatigue." | | | | | | | | | | | | | | | | |  |
| B3 | "Although I gave you this workout plan, you *should* adjust the speed/cadence/load/stretch/rest slightly whenever you feel it is uncomfortable or unpleasant."; "The goal is to perform 10-12 reps, but you can stop before if you feel it is becoming unpleasant."; "During this routine, adjust the speed to make you feel good during the exercise!" | | | | | | | | | | | | | | | | |  |
|  |  |  |  |  |  |  |  |  |  |  |  |  |  |  |  |  |  |  |
|  |  |  |  |  |  |  |  |  |  |  |  |  |  |  |  |  |  |  |
| C1 | "In your usual workout session schedule, we also have the group class XWZ and the ABC activity, which suits your goals. Do any of these activities spark your curiosity?"; "I noticed that you were enjoying doing XWZ in your last workouts. Would you like me to incorporate more of these in your workouts?" | | | | | | | | | | | | | | | | |  |
|  |  |  |  |  |  |  |  |  |  |  |  |  |  |  |  |  |  |  |
| C2 | FS scores < 1; sequence of planned exercises/routines with short intervals and higher intensities; reports or signs of considerable discomfort during specific activities | | | | | | | | | | | | | | | | |  |
| C3 | Plan session to promote a "ramping down" intensity; reduce intensity in the last minutes; choose lower activation activities for the end session | | | | | | | | | | | | | | | | |  |
| C4 | "In our class today, we will do XWZ and ABC, with 3 rounds and 20s intervals. How do you think this will make you feel (possibility to use an EVS scale)?" | | | | | | | | | | | | | | | | |  |
