# Supplementary material for: Impact of two brief behavioral theory–driven professional training programs on fitness center attendance: protocol for pragmatic controlled intervention with random allocation
Source: Front Psychol. 2026 Jul 2;17:1856891. doi: 10.3389/fpsyg.2026.1856891 (PMC13372327; doi:10.3389/fpsyg.2026.1856891)
Supplement: Supplementary file 6 [file Table_4.docx]

Supplementary file 2a - Single items proposal for professionals' intensity preference/tolerance assessment and agreement identification (PRETIE-Q) and enjoyment (PACES)

| Constructs | Single-item per construct |  |  |  |  |  |  |  |  |  |  |
| --- | --- | --- | --- | --- | --- | --- | --- | --- | --- | --- | --- |
| Preference | "While exercising, I prefer activities that are slow-paced and do not require much exertion." (item 7) | | | | | | | |  |  |  |
| Tolerance | "During exercise, if my muscles begin to burn excessively or if I find myself breathing very hard, it is time for me to ease off." (item 3) | | | | | | | | | | |
| Preference agreement | "Is the intensity of your training in accordance with your preference?" | | | | | | | | | |  |
| Tolerance agreement | "Is the intensity of your training in accordance with your tolerance?" | | | | | | | | | |  |
| Enjoyment | "Regarding my last exercise session, I enjoyed it/hated it." (item 1) | | | | |  |  |  |  |  |  |
